# Remoção Percutânea de Eletrodos de Estimulação Cardíaca Artificial em um Único Centro Sul-Americano

**DOI:** 10.36660/abc.20190726

**Published:** 2021-05-06

**Authors:** Bruna Costa Lemos Silva Di Nubila, Gustavo de Castro Lacerda, Helena Cramer Veiga Rey, Rodrigo Minati Barbosa

**Affiliations:** 1 Instituto Nacional de Cardiologia Rio de JaneiroRJ Brasil Instituto Nacional de Cardiologia, Rio de Janeiro, RJ - Brasil; 2 Hospital Pró-Cardíaco Rio de JaneiroRJ Brasil Hospital Pró-Cardíaco, Rio de Janeiro, RJ - Brasil

**Keywords:** Marca-Passo Artificial, Ressincronizador Cardíaco, Eletrodos Implantáveis

## Abstract

**Fundamento:**

Nas últimas décadas, o número de dispositivos eletrônicos cardíacos implantáveis (DCEI) aumentou consideravelmente, assim como a necessidade de remoção destes. Neste contexto, a remoção percutânea apresenta-se como uma técnica segura e capaz de evitar uma cirurgia cardíaca convencional.

**Objetivos:**

Primário: descrever a taxa de sucesso e complicações da remoção percutânea de DCEI em um hospital público brasileiro. Secundário: estabelecer preditores de sucesso e complicações.

**Métodos:**

Serie de casos retrospectiva de todos os pacientes submetidos à remoção de DCEI em um hospital público brasileiro no período de janeiro de 2013 a junho de 2018. Remoção, explante e extração de eletrodos, complicações e desfechos foram definidos conforme a diretriz norte-americana de 2017. Variáveis categóricas foram comparadas pelos testes Qui-quadrado ou Fisher, enquanto variáveis contínuas, por testes não pareados. O nível de significância adotado nas análises estatísticas foi de 5%.

**Resultados:**

61 pacientes foram submetidos à remoção de DCEI, sendo 51 extrações e 10 explantes. No total, 128 eletrodos foram removidos. Taxa de sucesso clínico foi 100% no grupo do explante e 90,2% no da extração (p=0,58). Complicações maiores foram encontradas em 6,6% dos pacientes. Falha do procedimento foi associada a eletrodos de ventrículo (p=0,05) e átrio (p=0,04) direito implantados há mais tempo. Duração do procedimento (p=0,003) e necessidade de transfusão sanguínea (p<0,001) foram associadas a maior índice de complicação.

**Conclusão:**

As taxas de complicação e sucesso clínico observadas foram de 11,5% e 91,8%, respectivamente. Remoções de eletrodos atriais e ventriculares mais antigos estiveram associados a menores taxa de sucesso. Procedimentos mais longos e necessidade de transfusão sanguínea foram associados a complicações.

## Introdução

Na última década, a ampliação das indicações de dispositivos cardíacos eletrônicos implantáveis (DCEI) e o envelhecimento da população aumentaram consideravelmente o número de pessoas com esses dispositivos.^1^^–^^5^ O número de eletrodos por paciente seguiu a mesma tendência devido à ampliação da indicação de terapia de ressincronização/desfibrilação cardíaca, *upgrades* e maior proporção de implante de dispositivos de dupla câmara em comparação ao número de implantes de dispositivo de câmara única.^3^^–^^6^

Situações em que a completa remoção dos DCEI é necessária, como no caso de complicações infecciosas ou vasculares, são frequentemente vistas atualmente.^5^^,^^7^^–^^9^ Desde 1980, novas técnicas e ferramentas vêm sendo desenvolvidas, permitindo uma extração percutânea segura desses dispositivos.^7^^,^^10^^–^^19^

No Brasil, o número de internações para implante de DCEI aumentou na última década e, atualmente, é responsável por 11.000 internações por ano.^20^ Consequentemente, a remoção desses dispositivos também cresceu de 79 em 2008 para 151 em 2016.^20^ A taxa de extração mundial de DCEI também aumentou, sendo aproximadamente de 10.000 a 15.000 eletrodos ao ano.^21^^,^^22^

Contudo, estudos mostrando a experiência brasileira e também sul-americana nesse procedimento são escassos na literatura. Dessa forma, esse trabalho visa evidenciar como objetivo primário a taxa de sucesso e de complicações nas remoções de DCEI em um hospital público brasileiro. Como objetivo secundário, buscou-se descrever os fatores associados ao sucesso e às complicações desse procedimento.

## Metodologia

### Desenho do estudo

Série de casos retrospectiva de pacientes submetidos à remoção de DCEI em um hospital quaternário brasileiro.

### Critérios de inclusão

Pacientes submetidos à remoção de DCEI no período de janeiro de 2013 a junho de 2018 foram incluídos no estudo.

### Técnica do procedimento

Todos os procedimentos foram realizados pelo mesmo cirurgião cardíaco. A remoção por simples tração foi primeiramente tentada e, em caso de insucesso, as bainhas mecânicas Evolution ou Evolution RL da Cook Medical® (*Cook medical Inc*., Bloomington, EUA) eram usadas.

Após a remoção, o reimplante de um novo dispositivo era realizado em tempo único em sítio contralateral quando as hemoculturas eram negativas e os pacientes não apresentavam sinais de infecção sistêmica. Nos indivíduos com sinais de infecção sistêmica ou hemocultura positiva, um reimplante em um segundo tempo foi realizado. Neste último caso, um mínimo de 2 semanas de antibioticoterapia era realizado, a contar da primeira hemocultura negativa após a remoção.

### Definições

Remoção de eletrodos foi definida como remoção de eletrodos por qualquer técnica.^23^ Explante foi definido como remoção de eletrodos em que todos os eletrodos tinham menos de 1 ano do implante e foram removidos sem nenhuma ferramenta ou somente usando estiletes, enquanto extração quando pelo menos um eletrodo tinha mais de 1 ano do implante ou necessitou do uso de bainhas e/ou estiletes.^23^

Sucesso clínico foi definido como remoção de todo DCEI do espaço vascular ou retenção de uma pequena porção (<4cm), sem que isso afete negativamente o desfecho do procedimento, sendo sucesso completo definido para parcela desses casos em que houve a remoção completa de todo DCEI do espaço vascular.^23^ Falha foi definida como ausência de sucesso clínico ou completo, desenvolvimento de uma sequela permanente ou morte relacionada ao procedimento.^23^ Definimos complicações maiores como as que trouxeram risco iminente à vida do paciente ou levaram-no a óbito; enquanto as menores como as que necessitaram de intervenção médica, incluindo intervenções cirúrgicas menores, mas que não ocasionaram sequela ao paciente.^23^

A infecção de loja foi definida como a presença de hiperemia, calor, flutuação, edema, dor ou drenagem purulenta da loja do marca-passo.^24^ Extrusão do gerador foi definida como a erosão da unidade geradora e/ou do eletrodo pela pele, com a exposição destes, com ou sem sinais de infecção local.^23^ Infecção de loja com bacteremia foi definida como sinais de infecção local na loja associada a hemoculturas positivas.^22^ Endocardite foi definida pela presença de vegetações na ecocardiografia e/ou quando os critérios de Duke foram preenchidos.^24^

### Análise estatística

A normalidade das variáveis foi verificada pelo teste de Kolmogorov-Smirnov. As variáveis contínuas com distribuição normal foram expressas em forma de média e desvio padrão, sendo analisadas pelo teste não pareado de Student. As variáveis contínuas sem distribuição normal foram descritas através de mediana e intervalo interquartil, sendo comparadas usando o teste não pareado de Mann-Whitney. As variáveis categóricas foram representadas em frequências e porcentagens e foram comparadas usando o teste do Qui-quadrado de Pearson ou Fisher. O nível de significância adotado nas análises estatísticas foi de 5%. Todas as análises foram realizadas utilizando o programa R, versão 3.3.0 e 3.4.1.

### Ética

O estudo foi aprovado pelo Comitê de Ética local (Aprovação número 67765317.6.0000.5272).

## Resultados

**Figura 1:**
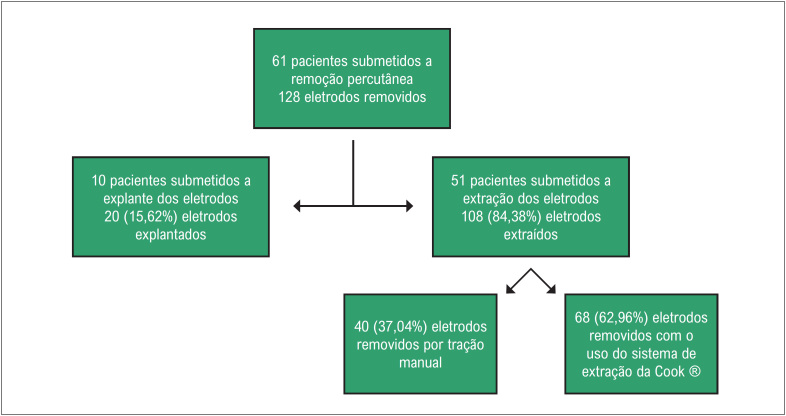
Seleção dos pacientes.

**Figura 2:**
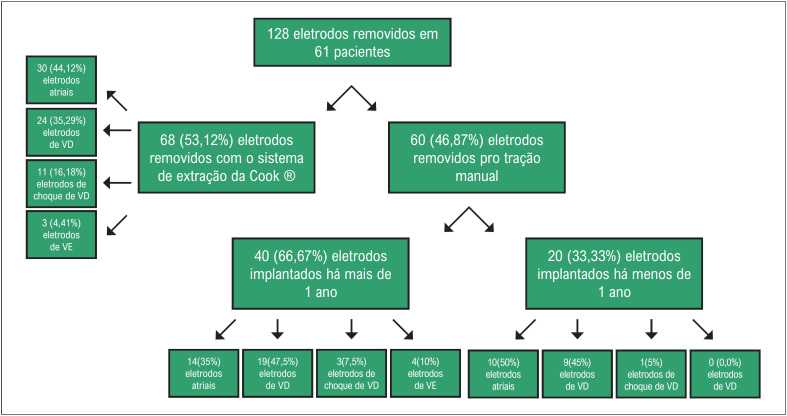
Tipos de eletrodos de estimulação cardíaca artificial. VD: ventrículo direito, VE: ventrículo direito

Os pacientes foram divididos conforme a Figura 1. As características demográficas dos pacientes foram discriminadas na Tabela 1. No grupo do explante, 11 (97,67%) dispositivos dupla câmara e 1 (8,33%) câmara única foram removidos, enquanto no da extração, 44 (89,8%) dupla câmara e 5 (10,2%) câmara única. A maior parte dos eletrodos nos dois grupos apresentava fixação ativa, apenas um eletrodo no grupo do explante (5%) e sete (6,5%) no grupo da extração apresentavam fixação passiva. Os tipos de eletrodos em cada grupo são apresentados na Figura 2.

**Tabela 1:** Características basais dos pacientes

	Explante (n=10)	Extração (n=51)	Valor de p
Sexo masculino n (%)	8 (80)	33 (64,7)	0,47
Idade (anos)	56,7 ± 25,64	60,63 ± 19,61	0,58
IMC (kg/m^2^)	21,43 ± 2,99	25,57 ± 4,15	0,02
**Testes laboratoriais**			
INR	1,15 [1,11 – 1,28]	1,1 [1,03 – 1,24]	0,18
Hemoglobina (g/dL)	12,5 [9,98 – 13,68]	12,5 [11,45 – 13,4]]	0,44
**Parâmetros ecocardiográficos**			
Fração de ejeção (%)	56,66 [47,42 – 66,45]	56,30 [31,2 – 64,3]	0,60
**Presença de insuficiência tricúspide n (%)**			0,12
Leve	5 (71,4)	13 (56,5)	
Moderada	0 (0,0)	7 (30,4)	
Grave	0 (0,0)	2 (8,7)	
**Comorbidades**			
Hipertensão arterial n (%)	7 (70,0)	30 (58,8)	1,0
Diabetes mellitus n (%)	1 (10,0)	16 (31,4)	0,26
Fibrilação atrial crônica n (%)	2 (20,0)	11 (21,6)	1,0
Doença cerebrovascular n (%)	0 (0,0)	2 (3,9)	1,0
Doença arterial coronariana n (%)	3 (30,0)	14 (27,5)	1,0
Insuficiência renal crônica n (%)	2 (20,0)	7 (13,7)	0,63
Anticoagulação n (%)	2 (20)	11 (21,6)	1,0
Cirurgia cardíaca prévia n (%)	4 (40,0)	15 (29,4)	0,71
**Idade dos eletrodos (meses)**			
Eletrodo atrial	3,73 [0,93 – 6,07]	83,6 [46,8 – 115,3]	<0,001
Eletrodo no ventrículo direito	3,73 [0,93 – 6,07]	87,9 [46,8 – 115,3]	<0,001
Eletrodo no seio coronariano	–	49,7 [29,4 – 83,6]	–

As variáveis contínuas foram representadas em médias ± desvio padrão e medianas ± intervalo interquatil. Variáveis categóricas foram representadas em frequências e porcentagens. Os p valores da tabela se referem aos testes de Student ou de Mann-Whitney para variáveis contínuas, teste do Qui-quadrado de Pearson ou de Fisher para variáveis categóricas. IMC: índice massa corporal; INR: international normalized ratio.

O motivo inicial para indicação dos 61 dispositivos foi bloqueio atrioventricular (BAV) total em 27 pacientes (44,3%), doença do nódulo sinusal em 5 (8,2%), BAV de 2° 2:1 fixo em 5 (8,2%), taquicardia ventricular (TV) sustentada com disfunção ventricular grave em 4 (6,6%), TV não sustentada com disfunção ventricular grave em 2 (3,3%), prevenção primária de morte súbita (MS) em paciente com cardiomiopatia hipertrófica em 2 (3,3%), BAV de 2° grau em 1 (1,6%), prevenção primária de MS na cardiomiopatia arritmogênica do ventrículo direito (VD) em 1 (1,6%), MS em 1 (1,6%), outras causas em 5 (8,2%) e desconhecida em 8 (13,1%). Quarenta pacientes (65,6%) tiveram o seu dispositivo cardíaco implantado em nosso hospital, enquanto 21 (34,42%), em outro.

Um total de 128 eletrodos foi removido desses 61 pacientes. Estes 61 pacientes foram submetidos ao procedimento cronologicamente da seguinte maneira: seis casos em 2013, nove em 2014, 18 em 2015, 12 em 2016, seis em 2017 e 11 nos primeiros 6 meses de 2018. As características do procedimento estão explicitadas na Tabela 2. Todos os pacientes do grupo do explante foram previamente submetidos a um implante de um novo DCEI, enquanto, no grupo da extração, 54,9% (28/51) foram submetidos previamente à troca de unidade geradora, 41,2% (21/51) a um novo implante e, em 2% (2/51), o procedimento prévio é desconhecido.

**Tabela 2:** Descrição do procedimento

	Explante (n=10)	Extração (n=51)	p valor
**Razão para remoção do dispositivo**			
Eletrodo disfuncionante n (%)	0 (0,0)	8 (15,7)	0,33
Endocardite relacionada ao marca-passo n (%)	3 (30)	14 (27,5)	1,0
Extrusão do marca-passo n (%)	0 (0,0)	12 (23,5)	0,19
Infecção de loja n (%)	1 (10)	10 (19,6)	0,67
*Upgrade* n (%)	0 (0,0)	1 (2)	1,0
Infecção de loja + bacteremia n (%)	4 (40)	5 (9,8)	0,09
**Número de eletrodos removidos por paciente**			0,75
1 n (%)	2 (20)	8 (15,7)	
2 n (%)	8 (80)	34 (66,7)	
3 n (%)	0 (0,0)	7 (13,7)	
4 n (%)	0 (0,0)	1 (2,0)	
5 n (%)	0 (0,0)	0 (0,0)	
6 ou mais n (%)	0 (0,0)	1 (2,0)	
**Desfecho**			0,58
Sucesso clínico n (%)	10 (100)	46 (90,2)	
Falha do tratamento n (%)	0 (0,0)	5 (9,8)	
Óbito n (%)	0 (0,0)	2 (3,9)	
Complicações n (%)	0 (0,0)	7 (13,7)	1,0
**Momento da complicação**			1,0
Periprocedimento n (%)	0 (0,0)	4/7 (57,1)	
Pós-procedimento n (%)	0 (0,0)	3/7 (42,9)	
**Tipo de complicação**			1,0
Maior n (%)	0 (0,0)	4/7 (57,1)	
Menor n (%)	0 (0,0)	3/7 (42,9)	
Transfusão sanguínea n (%)	0 (0,0)	5 (9,8)	0,58
Implante de novo dispositivo no mesmo dia da remoção n (%)	2 (22,2)	20 (55,6)	0,14
Implante de novo dispositivo após a remoção n (%)	9 (90,0)	36 (70,6)	0,27
Dias de hospitalização antes do procedimento	8 [5,25 – 22,0]	9,0 [4,0 – 17,5]	0,88
Dias de hospitalização após o procedimento	23 [6,0 – 63,0]	10,0 [4,0 – 23,5]	0,16

As variáveis contínuas foram representadas em médias ± desvio padrão e medianas ± intervalo interquatil. Variáveis categóricas foram representadas em frequências e porcentagens. Os p valores para variáveis contínuas se referem aos testes de Student ou de Mann-Whitney, para variáveis categóricas, teste do Qui-quadrado de Pearson ou de Fisher.

Na Tabela 2 é possível notar que causas infecciosas foram as mais comuns para remoção dos DCEI. Maior número de eletrodos removidos por paciente foi encontrado no grupo da extração. Dos pacientes com falha do procedimento, dois apresentaram óbito durante o procedimento devido à laceração do átrio direito e da veia cava superior, e foram incluídos nas complicações maiores. Entre os outros três pacientes, um deles teve a remoção indicada por infecção de loja, outro por extrusão de unidade geradora e um por necessidade de *upgrade* do eletrodo de VD. Um dos pacientes submetidos à extração com sucesso completo faleceu 5 dias após o procedimento devido a endocardite bacteriana e choque séptico. Entre os que tiveram sucesso clínico, 10 pacientes (100%) no grupo do explante e 38 (74,5%) no da extração tiveram sucesso completo do procedimento. As taxas de sucesso clínico e completo no total da população foram de 91,8% e 78,7%, respectivamente. A maioria dos pacientes foi submetida ao implante de um novo dispositivo.

Complicações e transfusão sanguínea ocorreram apenas no grupo de pacientes submetido à extração. O total de complicações encontrado foi 11,5%, sendo as complicações maiores responsáveis por 6,6%. Entre as complicações maiores, todas foram intraprocedimento, sendo dois óbitos, uma perfuração de VD corrigida cirurgicamente e uma parada cardiorrespiratória após remoção do eletrodo de VD com recuperação completa da paciente após manobras de reanimação cardiopulmonar. Todas as três complicações menores foram devido à hematoma de loja no pós-operatório com necessidade de drenagem cirúrgica. Todos estes três pacientes faziam uso de anticoagulantes, sendo dois usuários de varfarina e 1 de dabigatrana, os quais foram interrompidos com meia-vida adequada e/ou normalização da *international normalized ratio* (INR) antes do procedimento.

Entre os 21 pacientes com hemocultura positiva antes da remoção do dispositivo, os gram-positivos foram os mais comuns, estando presentes em 15 pacientes (93,8%) no grupo da extração e em quatro (80%) no grupo do explante. *S. aureus* foi a bactéria mais comum nos dois grupos em oito (50%) pacientes no da extração e quatro (80%) no do explante. Foi seguido pelo *S. epidermitis* e outros *Staphylococci* coagulase negativos.

Na Tabela 3, as variáveis associadas à falha do procedimento foram eletrodo atrial (p=0,04) e do VD (p=0,05) mais antigos. Na Tabela 4, a necessidade de transfusão sanguínea (p<0,001) e a duração do procedimento (p=0,003) revelaram-se associadas a maior índice de complicações.

**Tabela 3:** Fatores associados ao sucesso do procedimento

	Falha (n=5)	Sucesso clínico (n=56)	p valor
Sexo masculino n (%)	3 (60%)	38 (67,9%)	1,0
Idade (anos)	56,4 ±13,7	60,3 ± 21,08	0,69
Fração de ejeção ≤ 30% n (%)	2 (40)	8 (14,3)	0,44
**Comorbidades**			
Doença arterial coronariana n (%)	0 (0,0)	17 (30,4)	0,35
Diabetes mellitus n (%)	1 (20)	16 (28,6)	1,0
Insuficiência renal crônica n (%)	0 (0,0)	9 (16,1)	0,75
Presença de cirurgia torácica prévia n (%)	2 (40)	17 (30,4)	1,0
Remoção de eletrodo prévia n (%)	0 (0,0)	4 (7,1)	0,51
**Razão para remoção do eletrodo**			
Eletrodo disfuncionante n (%)	0 (0,0)	8 (14,3)	0,83
Endocardite relacionada ao marca-passo n (%)	2 (40)	15 (26,8)	0,91
Extrusão n (%)	0 (0,0)	12 (21,4)	0,57
Infecção de loja n (%)	2 (40)	9 (16,1)	0,47
Upgrade n (%)	1 (20)	0 (0,0)	0,12
Infecção de loja + bacteremia n (%)	0 (0,0)	9 (16,1)	0,58
**Número de eletrodos por paciente**			0,61
1 n (%)	2 (40)	8 (14,3)	
2 n (%)	3 (60)	38 (69,6)	
3 n (%)	0 (0,0)	7 (12,5)	
4 n (%)	0 (0,0)	1 (1,8)	
6 ou mais n (%)	0 (0,0)	1 (1,8)	
**Tipo de procedimento**			0,69
Explante n (%)	0 (0,0)	10 (17,9)	
Extração n (%)	5 (100)	46 (82,1)	
**Tipo do eletrodo removido**			
Atrial n (%)	5 (100)	51 (91,1)	1,0
Ventrículo direito n (%)	4 (80)	46 (82,1)	1,0
Eletrodo de choque do ventrículo direito n (%)	1 (20)	11 (19,6)	1,0
Eletrodo de seio coronariano n (%)	1 (20)	9 (16,1)	1,0
Idade do eletrodo atrial (anos)	9,5 [7,9 – 15,2]	5,1 [1,4 – 8,2]	0,04
Idade do eletrodo do ventrículo direito (anos)	9,5 [7,9 – 15,2]	5,1 [1,4 – 8,9]	0,05
Hemocultura positiva n (%)	2 (40,0)	19 (33,9)	0,89
Presença de *S. aureus* na hemocultura n (%)	1 (20)	11 (19,6)	1,0
Dias internado antes da remoção	13,0 [6,0 – 19,0]	8,5 [4,0 – 17,5]	0,34

As variáveis contínuas foram representadas em médias ± desvio padrão e medianas ± intervalo interquatil. Variáveis categóricas foram representadas em frequências e porcentagens. Os p valores para variáveis contínuas se referem aos testes de Student ou de Mann-Whitney, para variáveis categóricas teste do Qui-quadrado de Pearson ou de Fisher.

**Tabela 4:** Fatores associados a complicações no procedimento

	Com complicações (n=7)	Sem complicações (n=54)	p valor
Sexo masculino n (%)	4 (57,1)	37 (68,5)	0,67
Idade (anos)	50,14 ± 14,99	61,26 ± 20,9	0,18
Hemoglobina (g/dl)	11,5 [10,35 – 12,70]	12,7 [11,3 – 13,4]	0,34
INR	1,22 [1,17 – 1,29]	1.10 [1,04 – 1,24]	0,17
Fração de ejeção ≤ 30% n (%)	2 (28,6)	8 (14,8)	0,78
Presença de insuficiência tricúspide n (%)	4 (57,1)	26 (52,0)	1,0
**Comorbidades**			
Doença arterial coronariana n (%)	0 (0,0)	17 (31,5)	0,18
Diabetes mellitus n (%)	0 (0,0)	17 (31,5)	0,18
Insuficiência renal crônica n (%)	0 (0,0)	9 (16,7)	0,58
Anticoagulação n (%)	4 (57,1)	9 (16,7)	0,07
Presença de cirurgia cardíaca prévia n (%)	4 (57,1)	15 (27,8)	0,19
Remoção de eletrodo prévia n (%)	0 (0,0)	4 (7,4)	0,74
**Razão para remoção do eletrodo**			
Eletrodo disfuncionante n (%)	0 (0,0)	8 (14,8)	0,58
Endocardite relacionada ao marca-passo n (%)	2 (28,6)	15 (27,8)	1,0
Extrusão n (%)	2 (28,6)	10 (18,5)	0,62
Infecção de loja n (%)	3 (42,9)	8 (14,8)	0,10
Upgrade n (%)	0 (0,0)	1 (1,9)	1,0
Infecção de loja + bacteremia n (%)	0 (0,0)	9 (16,7)	0,58
**Número de eletrodos por paciente**			1,0
1 n (%)	1 (14,3)	10 (18,5)	
2 n (%)	5 (71,4)	36 (66,7)	
3 n (%)	1 (14,3)	6 (11,1)	
4 n (%)	0 (0,0)	1 (1,9)	
6 ou mais n (%)	0 (0,0)	1 (1,9)	
**Tipo de procedimento**			0,59
Explante n (%)	0 (0,0)	10 (18,5)	
Extração n (%)	7 (100)	44 (81,5)	
**Tipo do eletrodo removido**			
Atrial n (%)	6 (85,7)	50 (92,6)	0,47
Ventrículo direito n (%)	6 (85,7)	44 (81,5)	1,0
Eletrodo de choque n (%)	1 (14,3)	11 (20,4)	1,0
Eletrodo de seio coronariano n (%)	2 (28,6)	8 (14,8)	0,32
Idade do eletrodo atrial (anos)	7,7 [5,1 – 18,1]	5,1 [1,3 – 8,3]	0,16
Idade do eletrodo de ventrículo direito (anos)	8,1 [5,5 – 15,4]	5,2[1,4 – 8,7]	0,11
Idade do eletrodo de ventrículo esquerdo (anos)	3,9 [3,8 – 4,0]	5,2 [2,3 – 7,6]	0,77
Transfusão sanguínea n (%)	5 (71,4)	0 (0,0)	<0,001
Presença de *S. aureus* na hemocultura n (%)	1 (14,3)	11 (20,4)	1,0
Hemocultura positiva (%)	1 (14,3)	20 (37,0)	0,37
Duração do procedimento (minutos)	180 [146,25 – 202,50]	72,5 [47,75 – 105,0]	0,003
Dias internado antes da remoção	13 [6,5 – 29,5]	8 [4,00 – 15,50]	0,24

As variáveis contínuas foram representadas em médias desvio padrão e medianas ± intervalo interquatil. Variáveis categóricas foram representadas em frequências e porcentagens. Os p valores para variáveis contínuas se referem aos testes de Student ou de Mann-Whitney, para variáveis categóricas teste do Qui-quadrado de Pearson ou de Fisher. INR: international normalized ratio.

## Discussão

A idade média nos dois grupos evidencia uma população de meia-idade, porém com alto índice de comorbidades que acreditamos ter contribuído para o número elevado de complicações infecciosas relacionadas aos marca-passos. O número elevado de comorbidades cardiovasculares na população estudada também é compatível com pacientes de um centro altamente especializado em cardiologia em que um percentual considerável de pacientes havia sido submetido a uma cirurgia cardíaca previamente para reparo valvar ou revascularização miocárdica. Apesar de eletrodos mais antigos e a presença de comorbidades serem descritos por Sohail et al. como associados a maiores taxas de complicação, isso não foi confirmado nesse estudo.^24^

De acordo com Kusumoto et al. e Sohail et al., mulheres com infecção de dispositivo têm maior risco de óbito que homens.^23^^,^^24^ Contudo, no presente estudo, todos os óbitos ocorreram entre pacientes do sexo masculino. No presente estudo, o grupo da extração teve um maior número de eletrodos de choque do VD que o grupo do explante. Sohail et al. também relatam que esses eletrodos têm menor taxa de sucesso de extração por tração manual, sendo mais frequente a necessidade do uso de bainha extratora.^24^

Nesse estudo, todos os pacientes portadores de três ou mais eletrodos foram submetidos à extração, confirmando que quanto maior o número de eletrodos por paciente, maior a chance de se necessitar do uso de bainha extratora. Sohail et al. justificam tal achado devido ao aumento da quantidade de aderências com o aumento do número de eletrodos.^24^ Isso também é válido para associação de eletrodos de atriais e de VD mais antigos e falhas de extração percutânea.

As taxas de complicações maiores (6,6%) e de óbitos (3,3%) foram ligeiramente maiores no presente estudo do que nos centros de baixo volume (menos de 30 procedimentos ao ano) do registro ELECTRA (4,1% e 2,5%), o qual é o maior registro mundial de remoção de DCEI.^25^ Essa diferença pode ser atribuída à amostra mais reduzida do nosso estudo quando comparada à amostra do estudo ELECTRA, visto que os nossos valores são próximos aos encontrados neste estudo. Em relação às complicações menores (4,9%), esta foi bem próxima ao encontrado nesse mesmo registro (5,0%).^25^

A transfusão sanguínea, como esperado, esteve presente mais frequentemente entre os pacientes que apresentaram alguma complicação, visto que era necessária como suporte terapêutico em determinados casos. A maior duração do procedimento também esteve associada a maior taxa de complicações, semelhante ao observado nos centros de baixo volume do estudo ELECTRA em que houve maior duração do procedimento e de complicações em comparação aos centros de alto volume.^25^

A taxa de sucesso clínico na remoção de eletrodos (91,8%) foi ligeiramente menor que nos centros de baixo volume do estudo ELECTRA (94,3%), o que também pode ter sido influenciado pelo tamanho amostral.^25^ Bongiorni et al., recentemente, relatam a sua experiência em um centro de alto volume europeu com uma taxa de sucesso completo de 98,4% (2015).^26^ Esta é muito maior que a observada nesse estudo (78,7%) o qual é bem próximo ao observado por Eckhard et al. (81%, 1996).^27^ Neste último estudo, a taxa de falha na remoção do sistema foi semelhante à encontrada na nossa população (7% *vs*. 8%).^27^ No ELECTRA, a tração manual esteve mais presente nos centros de baixo volume, o que é compatível com a nossa proporção de tração manual.^25^

O número de dias dentro do hospital depois da remoção no grupo do explante foi maior que no da extração (10 *vs*. 23), pois mais da metade dos pacientes do primeiro grupo (70%) tinha endocardite ou infeção de loja com hemoculturas positivas, o que foi menos observado no segundo grupo (37,3%). Isso é compatível com o achado de que todos os eletrodos explantados foram precedidos do implante de um novo DCEI, sendo a bacteremia durante o implante a mais provável causa. Dessa forma, o grupo do explante necessitou de maior tempo de antibioticoterapia após a remoção e, consequentemente, maior tempo de hospitalização no pós-operatório.

Kutarski et al. e Bongiorni et al. relatam que a avulsão cardíaca é mais comum em centros que usam a bainha mecânica quando comparada à avulsão vascular.^26^^,^^28^ Isso foi confirmado nesse artigo, visto que a avulsão cardíaca foi encontrada no dobro de pacientes quando comparada à avulsão vascular. No paciente que foi a óbito por avulsão vascular, nenhuma oclusão vascular foi documentada, a qual Zucchelli et al. descrevem como fator prognóstico para esse tipo de complicação.^29^ Esses mesmos autores associam a presença do eletrodo de choque St Jude Medical Riata^®^ (St. Jude Medical, Inc., St. Paul, MN, EUA) e presença de três ou mais eletrodos com maiores índices de avulsão cardíaca.^29^ Contudo, os pacientes que apresentaram essa complicação em nossa população apresentavam dois eletrodos, um atrial e outro ventricular direito, e este último não era um eletrodo de choque.

Este estudo apresenta algumas limitações que precisam ser consideradas. Além de ser de um único centro, foi uma análise retrospectiva e, por isso, alguns eventos podem não ter sido reportados. Esse estudo também retrata a experiência inicial do uso de bainha mecânica em nosso hospital, referindo-se a uma curva de aprendizado, o que pode ter refletido em um menor percentual de sucesso e maiores índices de complicações. O número de extrações durante o período designado pode não ter alcançado o observado em outros estudos europeus e americanos, os quais reportam mais de mil pacientes em algumas series. Contudo, é um número considerável quando comparado à realidade dos países latino-americanos em que poucos artigos evidenciando a experiência sul-americana são encontrados na literatura e, entre os poucos publicados, estes apresentam menos de 40 pacientes com uso de bainha mecânica.

## Conclusão

A taxa de complicação total observada foi de 11,5%, e a taxa de sucesso clínico foi de 91,8%. Remoções de eletrodos atriais e ventriculares mais antigos estiveram associadas a menores taxas de sucesso. Apesar de procedimentos mais longos e necessidade de transfusão sanguínea não serem a causa de maiores complicações, estes foram mais encontrados nesse grupo de pacientes.

Os resultados deste estudo reforçam que, mesmo em hospitais públicos brasileiros com recursos limitados e, consequentemente, menor volume de extrações ao ano, é possível alcançar sucesso no procedimento na maioria dos pacientes submetidos à remoção percutânea dos DCEI e com taxas de sucesso e complicações próximas às encontradas aos centros de baixo volume europeus.
