# Supplementary material for: Remoção Percutânea de Eletrodos de Estimulação Cardíaca Artificial em um Único Centro Sul-Americano
Source: Arq Bras Cardiol. 2021 May 6;116(5):908–16. [Article in Portuguese] doi: 10.36660/abc.20190726 (PMC8121483; doi:10.36660/abc.20190726)
Supplement: Translation

# Translation: Percutaneous Removal of Cardiac Leads in a Single Center in South America

**Authors:** Bruna Costa Lemos Silva Di Nubila, Gustavo de Castro Lacerda, Helena Cramer Veiga Rey, Rodrigo Minati Barbosa

**Affiliations:** 1 Rio de JaneiroRJ Brazil Instituto Nacional de Cardiologia, Rio de Janeiro, RJ - Brazil; 2 Rio de JaneiroRJ Brazil Hospital Pró-Cardíaco, Rio de Janeiro, RJ - Brazil

**Keywords:** Artificial Pacemaker, Cardiac Resynchronization Therapy, Implantable Electrodes

## Abstract

**Background:**

In the last decade, the number of cardiac electronic devices has risen considerably and consequently the occasional need for their removal. Concurrently, the transvenous lead removal became a safe procedure that could prevent open-heart surgery.

**Objective:**

The primary objective of this study was to describe the successful performance and the complication rates of pacemaker removals in a Brazilian public hospital. Our secondary aim was to describe the variables associated to successes and complications.

**Methods:**

A retrospective case series was conducted in patients submitted to pacemaker removal in a Brazilian public hospital from January 2013 to June 2018. Removal, explant, extraction, success and complication rates were defined by the 2017 Heart Rhythm Society Guideline. Categorical variables were compared using x^2^ or Fisher's tests, while continuous variables were compared by unpaired tests. A p-value of 0.05 was considered statistically significant.

**Results:**

Cardiac device removals were performed in 61 patients, of which 51 were submitted to lead extractions and 10 to lead explants. In total, 128 leads were removed. Our clinical success rate was 100% in the explant group and 90.2% in the extraction one (p=0.58). Major complications were observed in 6.6% patients. Procedure failure was associated to older right ventricle (p=0.05) and atrial leads (p=0,04). Procedure duration (p=0.003) and need for blood transfusion (p<0,001) were associated to more complications.

**Conclusion:**

Complications and clinical success were observed in 11.5% and 91.8% of the population, respectively. Removal of older atrial and ventricular leads were associated with lower success rates. Longer procedures and blood transfusions were associated with complications.

## Introduction

In the past decade, the prevalence of cardiac implantable electronic devices (CIEDs) have increased due to broader indications and population aging.^1^^–^^5^ The number of leads per patient has also increased, with more indications of cardiac resynchronization/defibrillator therapy, upgrades and a higher proportion of dual vs. single-chamber devices.^3^^–^^6^

Despite the evolution of CIEDs, situations which require complete device and lead removal, such as infections and vascular complications, are still observed.^5^^,^^7^^–^^9^ Since 1980, new techniques and tools have been developed to allow safe percutaneous removal of these devices.^5^^,^^10^^–^^19^

In Brazil, the number of hospital admissions for CIEDs implant has increased over the last decades and currently there are 11,000 hospitalizations per year.^20^ Consequently, hospital admissions to remove these CIEDs increased from 79 hospitalizations in 2008 to 151 in 2016.^20^ Worldwide, the annual rate of CIED extraction has increased, ranging from 10,000 to 15,000 leads per year.^21^^,^^22^

Data from Brazilian and the South American experience in percutaneous leads extraction are lacking in the literature. Thus, the primary goal of our study was to describe the success and complication rates in CIED removals at a Brazilian public hospital. Additionally, we described the variables associated with procedure success and complications.

## Metodology

### Study Design

We performed a retrospective study in patients submitted to CIED removal at a Brazilian quaternary hospital.

### Inclusion criteria

All patients with the recommendation of CIED removal from January 2012 to June 2018 were included.

### Procedure technique

All procedures were performed by the same cardiac surgeon. Simple retraction was attempted first and, if not successful, the Evolution or the Evolution RL mechanical sheaths by Cook Medical® (Cook medical Inc., Bloomington, [USA]) was used.

Reimplantation was performed as a one-step approach on the contralateral side in patients without systemic infection or positive blood cultures. In patients with elevated infection markers or positive blood cultures, a second-step approach was performed. In the latter case, antibiotic therapy was carried out for a minimum of 2 weeks after the first negative blood culture.

### Definitions

Lead removal was defined as lead removal by any technique.^23^ Lead explant was defined as the lead removal procedure where all leads were removed without tools or with stylets only and all removed leads had < 1 year since the implant.^23^ Extraction was defined as the lead removal procedure where at least one lead required the assistance of equipment not typically employed during the implant or at least one lead had been implanted for > 1 year.^23^

Clinical success was defined as the lead extraction procedure with the removal of all target lead material from the vascular space or the retention of a small portion of the lead (<4 cm) that does not negatively impact the procedure outcome.^23^ Those in this group who had a complete removal of all target leads and lead material from the vascular space were named as complete procedural success.^23^ Failure was defined as the lead extraction procedure in which complete procedural or clinical success could not be achieved, or as the developmentof any permanent disability or procedure-related death.^23^ Major complications were the ones that posed an imminent risk of death or resulted in death, while minor ones were undesired adverse events that required medical intervention, including minor intervention, but did not significantly affect patient's functions.^23^

Pocket infection was defined as the presence of erythema, warmth, fluctuation, edema, pain or purulent drainage from the device pocket.^24^ Isolated pocket erosion was defined as device and/or lead(s) eroding through the skin, with exposure of the generator or leads, with or without local signs of infection.^23^ Pocket site infection with bacteremia was defined as local infection signs and positive blood cultures.^22^ Endocarditis was defined as the presence of vegetation in the echocardiogram and/or when Duke criteria were met.^24^

### Statistical analysis

The normal distribution was verified with the Kolmogorov-Smirnov test. Continuous variables with normal distribution were expressed as mean and standard deviation and compared by unpaired Student's T-test. The ones with non-normal distribution were expressed as median and interquartile ranges and compared by the Mann–Whitney test. Categorical variables were presented as frequencies and percentages and were compared using x^2^ or Fisher's exact tests. A p-value of 0.05 was considered statistically significant. All statistical analyses were performed using the R program, version 3.3.0 and 3.4.1.

### Ethical approval

The study was approved by the local Research Ethics Committee (67765317.6.0000.5272).

## Results

The study flow chart is provided in Figure 1. Table 1 displays patient demographics. While the explant and extraction groups had 11 (97.67%) and 44 (89.80%) dual chamber devices, only 1 (8.33%) and 5 (10.2%) single-chamber devices were seen in each group, respectively. The majority of the leads had an active fixation, whereas only one lead (5%) in the explant and 7 (6.5%) in the extraction groups had passive fixation. Another flow chart with the lead types in each group is shown in Figure 2.

**Figure 1:** Patient selection.

**Table 1:** Patient Demographics

	Explant (n=10)	Extraction (n=51)	p-value
Male gender n(%)	8(80)	33(64.7)	0.47
Age (years)	56.7 ± 25.64	60.63 ± 19.61	0.58
BMI (Kg/m^2^)	21.43 ± 2.99	25.57 ± 4.15	0.02
**Blood tests**			
INR	1.15 [1.11 – 1.28]	1.1 [1.03 – 1.24]	0.18
Hemoglobin (g/dl)	12.5 [9.98 -13.68]	12.5 [11.45 -13.4]	0.44
**Echocardiographic features**			
EF (%)	56.66 [47.42 – 66.45]	56.30 [31.2 – 64.3]	0.6
**Presence of tricuspid regurgitation n(%)**			0.12
mild	5 (71.4)	13 (56.5)	
moderate	0 (0.0)	7 (30.4)	
severe	0 (0.0)	2 (8.7)	
**Comorbidities**			
Hypertension n(%)	7 (70.0)	30 (58.8)	1.0
Diabetes Mellitus n(%)	1 (10.0)	16 (31.4)	0.26
Chronic atrial fibrillation n(%)	2 (20.0)	11 (21.6)	1.0
Cerebrovascular disease n(%)	0 (0.0)	2 (3.9)	1.0
Coronary artery disease n(%)	3 (30.0)	14 (27.5)	1.0
Chronic kidney disease n(%)	2 (20.0)	7 (13.7)	0.63
Anticoagulation n(%)	2 (20.0)	11 (21.6)	1.0
Previous cardiac surgery n(%)	4 (40.0)	15 (29.4)	0.71
**Lead use (months)**			
Atrial leads	3.73 [0.93 – 6.07]	83.6 [46.8 – 115.3]	<0,001
Right ventricular leads	3.73 [0.93 – 6.07]	87.9 [46.8 – 115.3]	<0,001
Left ventricle leads	–	49.7[29.4 – 83.6]	–

Continuous variables were shown as mean ± standard deviation and median ± interquartile ranges. Categorical variables were presented as frequencies and percentages. P-values in the table are related to the Student's or Mann-Whitney test for continuous variables and x^2^ and Fisher Tests for categorical variables. BMI: body mass index; INR: International Normalized Ratio; EF: ejection fraction.

**Figure 2:** Leads type. RV: right ventricle; LV: left ventricle.

The primary implant indication was complete heart block in 27 patients (44.3%), sick-sinus disease in 5 (8.2 %), 2:1 second-degree heart block in 5 (8.2%), sustained ventricular tachycardia with severe ventricular disfunction in 4 (6.6%), non-sustained ventricular tachycardia with severe ventricular disfunction in 2 (3.3%), primary prevention in hypertrophic cardiomyopathy in 2 (3.3%), second-degree heart block in 1 (1.6%), primary prevention in arrhythmogenic right ventricular cardiomyopathy in 1 (1.6%), sudden cardiac arrest in 1 (1.6%), other reasons in 5 (8.2%) and unknow in 8 (13.1%) cases. Forty (65.6%) patients had the cardiac device implanted in our hospital, while 21 (34.42%) had the device implanted in another institution.

A total of 128 leads were removed from these 61 patients. Chronologically, patient procedures were distributed as follow: 6 in 2013, 9 in 2014, 18 in 2015, 12 in 2016, 6 in 2017 and 11 in the first six months of 2018. Procedure characteristics are displayed in Table 2. Before the removal, all patients from the explant group were submitted to a new pacemaker implant, whilst in the extraction group 54.9% (28/51) were submitted to a battery replacement, 41.2% (21/51) to a new implant and in 2% (2/51) the previous procedure was unknown.

**Table 2:** Procedure description

	Explant (n=10)	Extraction (n=51)	p-value
**Reason for device removal**			
Dysfunctional lead n(%)	0 (0.0)	8 (15.7)	0.33
Device-related endocarditis n(%)	3 (30)	14 (27.5)	1.0
Isolated pocket erosion n(%)	0 (0.0)	12 (23.5)	0.19
Pocket infection n(%)	1 (10)	10 (19.6)	0.67
Upgrade n(%)	0 (0.0)	1 (2)	1.0
Pocket infection with bacteremia n(%)	4 (40)	5 (9.8)	0.09
**Number of leads removed per patient**			0.75
1 n(%)	2 (20)	8 (15.7)	
2 n(%)	8 (80)	34 (66.7)	
3 n(%)	0 (0.0)	7 (13.7)	
4 n(%)	0 (0.0)	1 (2.0)	
5 n(%)	0 (0.0)	0 (0.0)	
6 or more n(%)	0 (0.0)	1 (2.0)	
**Outcome**			0.58
Clinical success n(%)	10 (100)	46 (90.2)	
Failure n(%)	0 (0.0)	5 (9.8)	
Death n(%)	0 (0.0)	2 (3.9)	
Complications n(%)	0 (0.0)	7 (13.7)	1.0
**Time of complication**			1.0
Intra-procedural n(%)	0 (0.0)	4/7 (57.1)	
Post-procedural n(%)	0 (0.0)	3/7 (42.9)	
**Type of complication**			1.0
Major n(%)	0 (0.0)	4/7 (57.1)	
Minor n(%)	0 (0.0)	3/7 (42.9)	
Blood transfusion n(%)	0 (0.0)	5 (9.8)	0.58
Implant of a new device on removal date n(%)	2 (22.2)	20 (55.6)	0.14
Implant of a new device after the removal n(%)	9 (90.0)	36 (70.6)	0.27
Days of hospitalization before the procedure n(%)	8 [5.5 – 22.0]	9.0 [4.0 – 17.5]	0.88
Days of hospitalization after the procedure n(%)	23 [6.0 – 63.0]	10.0 [4.0 – 23.5]	0.16

Continuous variables were shown as mean ± standard deviation and median ± interquartile ranges. Categorical variables were presented as frequencies and percentages. P-values in the table are related to the Student's or Mann-Whitney test for continuous variables and x^2^ and Fisher Tests for categorical variables.

Table 2 showed that infection was the most common reason for the device to be removed. More leads were removed in the extraction group. Among the failed procedures, 2 died because of right atrium and superior vena cava tears, which were considered major complications. The three other patients in this group had the removal indicated for pocket infection, lead extrusion and the need to upgrade the right ventricle (RV) lead. One patient with a completely successful procedure died 5 days later due to endocarditis and septic shock. Among those with clinical success, 10 (83.33%) and 38 (88.37%) in the explant and extraction group attained complete success rate, respectively. The overall clinical success rate was 91.8% and the overall complete success rate was 78.7%. Most patients were submitted to a new device implant.

Complications and blood transfusions were only observed in the extraction group. Major complication rate was 6.6% within a 11.5% overall rate of complications. All major complications were during the procedure, comprising 2 deaths; one RV perforation and one cardiac arrest following RV lead removal with full recover after cardiopulmonary resuscitation. All minor complications were due to pocket hematoma, which required a surgical approach. These three patients were taking anticoagulants, of which 2 were on Warfarin and 1 on Dabigatran. All anticoagulants were stopped with an adequate half-life and the INR was normalized prior to the procedure.

Of the 21 patients with positive blood cultures before lead removal, Gram-positive bacteria were more common in 15 patients (93.8%) in the extraction group and 4 (80%) in the explant one. *S. aureus* was the most common bacteria in both groups with 8 cases (50%) in the extraction group and 4 (80%) in the explant one. The second most common microorganism was *S. epidermitis,* followed by Coagulase-negative staphylococci.

Table 3 shows that the only variable associated with procedure failure was older right atrium (p=0.04) and RV (p=0.05) leads. Procedure failure was associated to an older right ventricle lead (p=0.018). Table 4 shows that the need for blood transfusion (p<0.001) and procedure duration (p=0.003) were associated with more complications.

**Table 3:** Variables associated with procedure success

	Failure (n=5)	Clinical success (n=56)	p-value
Male gender n(%)	3 (60)	38 (67.9)	1.0
Age (years)	56.4 ±13.7	60.3 ± 21.08	0.687
Ejection fraction ≤ 30% n(%)	2 (40)	8 (14.3)	0.444
**Comorbidities**			
Coronary artery disease n(%)	0 (0.0)	17 (30.4)	0.352
Mellitus diabetes n(%)	1 (20)	16 (28.6)	1.0
Chronic kidney disease n(%)	0 (0.0)	9 (16.1)	0.754
Previous thoracic surgery n(%)	2 (40)	17 (30.4)	1.0
Previous lead removal n(%)	0 (0.0)	4 (7.1)	0.513
**Reason for device removal**			
Dysfunctional lead n(%)	0 (0.0)	8 (14.3)	0.83
Device-related endocarditis n(%)	2 (40)	15 (26.8)	0.912
Isolated pocket erosion n(%)	0 (0.0)	12 (21.4)	0.57
Pocket infection n(%)	2 (40)	9 (16.1)	0.468
Upgrade n(%)	1 (20)	0 (0.0)	0.124
Pocket infection with bacteremia n(%)	0 (0.0)	9 (16.1)	0.579
**Number of leads removed per patient**			0.606
1 n(%)	2 (40)	8 (14.3)	
2 n(%)	3 (60)	38 (69.6)	
3 n(%)	0 (0.0)	7 (12.5)	
4 n(%)	0 (0.0)	1 (1.8)	
6 or more n(%)	0 (0.0)	1 (1.8)	
**Type of procedure**			0.687
Explant n(%)	0 (0.0)	10 (17.9)	
Extraction n(%)	5 (100)	46 (82.1)	
**Type of lead removed**			
Atrial n(%)	5 (100)	51 (91.1)	1.0
Right ventricle n(%)	4 (80)	46 (82.1)	1.0
Defibrillator lead n(%)	1 (20)	11 (19.6)	1.0
Left ventricle lead n(%)	1 (20)	9 (16.1)	1.0
Atrial lead age (years)	9.5 [7.9 – 15.2]	5.1 [1.4 – 8.2]	0.04
Right ventricle lead age (years)	9.5 [7.9 – 15.2]	5.1 [1.4 – 8.9]	0.05
Positive Blood culture n(%)	2 (40)	19 (33.9)	0.887
Presence of *S. aureus* in blood culture n(%)	1 (20)	11 (19.6)	1.0
Days in hospital before removal	13.0 [6.0 – 19.0]	8.5 [4.0 – 17.5]	0.343

Continuous variables were shown as mean ± standard deviation and median ± interquartile ranges. Categorical variables were presented as frequencies and percentages. P-values in the table are related to the Student's or Mann-Whitney test for continuous variables and x^2^ and Fisher Tests for categorical variables.

**Table 4:** Variables associated with procedure complications

	Presence of complications (n=7)	Absence of complications (n=54)	p-value
Male gender n(%)	4(57.1)	37(68.5)	0.67
Age (years)	50.14 ± 14.99	61.26 ± 20.9	0.18
Hemoglobin (g/dl)	11.5 [10.35 – 12.70]	12.7 [11.3 – 13.4]	0.34
INR	1.22 [1.17 – 1.29]	1.10 [1.04 – 1.24]	0.17
Ejection fraction ≤ 30% n(%)	2 (28.6)	8 (14.8)	0.78
Presence of tricuspid regurgitationn(%)	4 (57.1)	26 (52.0)	1.0
**Comorbidities**			
Coronary artery disease n(%)	0 (0.0)	17 (31.5)	0.18
Mellitus diabetes n(%)	0 (0.0)	17 (31.5)	0.18
Chronic kidney disease n(%)	0 (0.0)	9 (16.7)	0.58
Anticoagulation n(%)	4 (57.1)	9 (16.7)	0.07
Previous cardiac surgery n(%)	4 (57.1)	15 (27.8)	0.19
Previous lead removal n(%)	0 (0.0)	4 (7.4)	0.74
**Reason for device removal**			
Dysfunctional lead n(%)	0 (0.0)	8 (14.8)	0.58
Device-related endocarditis n(%)	2 (28.6)	15 (27.8)	1.0
Isolated pocket erosion n(%)	2 (28.6)	10 (18.5)	0.62
Pocket infection n(%)	3 (42.9)	8 (14.8)	0.1
Upgrade n(%)	0 (0.0)	1 (1.9)	1.0
Pocket infection with bacteremia n(%)	0 (0.0)	9 (16.7)	0.58
**Number of leads removed per patient**			1.0
1 n(%)	1 (14.3)	10 (18.5)	
2 n(%)	5 (71.4)	36 (66.7)	
3 n(%)	1 (14.3)	6 (11.1)	
4 n(%)	0 (0.0)	1 (1.9)	
6 or more n(%)	0 (0.0)	1 (1.9)	
**Type of procedure**			0.59
Explant n(%)	0 (0.0)	10 (18.5)	
Extraction n(%)	7 (100)	44 (81.5)	
**Type of lead removed**			
Atrial n(%)	6 (85.7)	50 (92.6)	0.47
Right ventricle n(%)	6 (85.7)	44 (81.5)	1.0
Defibrillator lead n(%)	1 (14.3)	11 (20.4)	1.0
Left ventricle lead n(%)	2 (28.6)	8 (14.8)	0.32
Atrial leads age (years)	7.7 [5.1 – 18.1]	5.1 [1.3 – 8.3]	0.16
Right ventricle lead age (years)	8.1 [5.5 – 15.4]	5.2 [1.4 – 8.7]	0.11
Left ventricle lead age (years)	3.9 [3.8 – 4.0]	5.2 [2.3 – 7.6]	0.77
Blood transfusion n(%)	5 (71.4)	0 (0.0%)	<0.001
Presence of *S. aureus* in blood culture n(%)	1 (14.3)	11 (20.4)	1.0
Positive blood culture n(%)	1 (14.3)	20 (37)	0.37
Procedure duration in minutes (minutes)	180 [146.25 – 202.5]	72.5 [47.75 – 105.0]	0.003
Days in hospital before removal	13 [6.5 – 29.5]	8 [4.0 – 15.5]	0.24

Continuous variables were shown as mean ± standard deviation and median ± interquartile ranges. Categorical variables were presented as frequencies and percentages. P-values in the table are related to the Student's or Mann-Whitney test for continuous variables and x^2^ and Fisher Tests for categorical variables. INR: International Normalized Ratio.

## Discussion

The overall age in both groups shows an older population with a high percentage of comorbidities, which we believe contributed to the high infection rate in the device-related procedures. Cardiovascular comorbidities were commonly seen, since our hospital is a quaternary center specialized in cardiology and a considerable percentage of patients have been submitted to a previous cardiac procedure, either valve surgery or coronary artery bypass grafting. Although Sohail et al. described older leads and comorbidities as associated with more complications, this could not be confirmed in this study.^24^

Kusumoto et al. and Sohail et al., found that women have a higher risk of death than men.^23^^,^^24^ However, in our study, both deaths were observed in male patients. We also found that the extraction group had more defibrillator leads than the explant group. Sohail et al. also stated that these leads show lower success rates with manual traction and that extraction sheaths are commonly needed in the procedure.^24^

In this study, all patients with three or more leads were submitted to an extraction, confirming that a higher number of leads per patient is associated to a higher risk of requiring the use of extraction sheaths. Sohail et al. stated that higher number of leads per patients is associated to more adherence, which could justify this finding.^24^ This is also true in cases of failure in removing older atrium and RV leads.

The rates of major complications (6.6%) and deaths (3.3%) were slightly higher when compared to the low-volume centers (less than 30 extractions per year) in the ELECTRA study (4.1% and 2.5%), which is the largest worldwide register of CIED removals.^25^ We believe that our smaller population might have contributed to this difference. Minor complication rates (4.9%) were similar to the rates in this registry (5.0%).^25^

As expected, blood transfusion was more frequent among patients with complications, since it was used as treatment in some cases. Longer procedures were associated with more complications. This reinforced the finding in the ELECTRA study, which showed that the low-volume centers had longer procedures and more complications when compared to the high-volume centers.^25^

Our clinical success rate (91.8%) was slightly lower than that described in the low-volume centers in the ELECTRA study (94.3%), probably because of the smaller number of enrolled patients.^25^ Recently, Bongiorni et al. showed their experience in a high-volume center in Europe, with 98.4% of complete procedural success (2015).^26^ This rate was dramatically higher than in our study (78.7%), but our figure is similar to that described by Eckhard A et al. (81% – 1996).^27^ They also had a similar failure rate when compared to ours (7% vs 8%).^27^ In the ELECTRA study, manual traction was more common in low-volume centers, which is compatible with our percentage of manual traction.^25^

The number of hospitalization days after the procedure in the explant group was more than double when compared to the extraction group (10 vs. 23 days), due to the fact that more than half of the patients in the former group (70% vs. 37.3%) had device-related endocarditis or pocket infection with bacteremia. The fact that all explant procedures were preceded by a pacemaker implant suggests that bacteremia during the implant was the most common reason for this finding. Hence, positive blood culture results were more frequently observed in the explant group, which was responsible for the longer hospital stay after the procedure to complete the antibiotic therapy.

Kutarski et al. and Bongiorni et al. stated that cardiac tears are more common than vascular tears in centers that use mechanical sheaths.^26^^,^^28^ This was also seen in this study, since the cardiac tear was present in twice the number of patients with vascular tear. The patient who died due to a vascular tear did not have a prior documented vascular occlusion, which is described as a prognostic factor for this complication by Zucchelli et al. ^29^ This same author states that the St Jude Medical Riata^®^ defibrillator (St. Jude Medical, Inc., St. Paul, MN, USA) leads and three or more leads were associated with cardiac tears.^29^ However, our two patients who had these complications had two leads, in the atrium and non-defibrillator RV leads.

Our study has some limitations which should be considered. This was a retrospective analysis; therefore, clinical events may have been underreported. This study has shown an initial experience with mechanical sheaths in our hospital and this learning curve could have contributed to a lower success and a higher major complication rates. The number of extraction procedures during the designated period did not reach the ones reported in the European and American studies, of which some have shown more than 1,000 patients. However, in South America, this is a significant number since fewer studies using mechanical sheaths have been published in which the sample had fewer than 40 patients.

## Conclusion

Our overall complication and clinical success rates were 11.5% and 91.8%, respectively. Older atrial and ventricular leads were associated to lower success rates. Although longer procedures and blood transfusions were more frequently seen in the complication group, these were not the reasons for complications.

Our results reaffirm that even in public Brazilian hospitals with limited resources and consequently, with lower extraction volumes per year, success can be achieved in the majority of the cases of transvenous lead extractions. Moreover, the success and complication rates were similar to the ones in low-volume centers in Europe.
